# Integrin α5β1 simultaneously controls EGFR-dependent proliferation and Akt-dependent pro-survival signaling in epidermoid carcinoma cells

**DOI:** 10.18632/aging.100457

**Published:** 2012-05-22

**Authors:** Galina E Morozevich, Nadezda I Kozlova, Natalia A Ushakova, Marina E Preobrazhenskaya, Albert E Berman

**Affiliations:** V.N. Orekhovich Institute of Biomedical Chemistry RAMS, 119121 Moscow, Russia

**Keywords:** integrins, EGFR, proliferation, apoptosis, signal transduction

## Abstract

To delineate distinctive role of the components of α5β1 integrin-EGFR axis in control of epidermoid carcinoma cell proliferation, we performed individual inhibition of α5β1 and EGFR via genetic and phamacological methods, respectively. We demonstrated that pharmacological inhibition of epidermal growth factor receptor (EGFR) significantly affected proliferation of A431 human cells by inducing the G0/G1 cell cycle arrest, whereas shRNA-mediated depletion of α5 subunit of α5β1 integrin led to a similar type of cell cycle arrest followed by significant apoptosis. Both treatments resulted in suppression of activated (phosphorylated) forms of focal adhesion kinase (FAK) and Erk. However, unlike EGFR inhibition, depletion of α5 led to substantial suppression of AKT activity. Accordingly, pharmacological inhibition of EGFR and AKT recapitulated detrimental effects caused by shRNA-mediated depletion of α5. Moreover, depletion of α5 led to a severe drop in the amounts of active EGFR. Thus, for the first time, we demonstrated that α5β1 integrin simultaneously maintains pro-survival signaling via continuous activation of AKT and up-regulates proliferation via activation of EGFR.

## INTRODUCTION

Cell proliferation is controlled by cytokines including growth factors and the components of extracellular matrix. Both types of proteins initiate signal transduction through growth factor specific receptors and matrix-specific receptors integrins, and their disbalance may lead to the uncontrolled proliferation and carcinogenesis [1-4]. Different integrins can interact with the same matrix proteins thus generating physiologically similar signals [5] making evaluation of the functional impact of individual integrins a cumbersome task.

The fibronectin-binding α5β1 is the only integrin with the single ligand specificity and yet, it often exerts controversial effects on cell proliferation and carcinogenesis ranging from stimulatory to inhibitory [6-10]. The mechanisms underlying the regulation of cell proliferation by integrins have not been completely characterized. One such mechanism includes interaction between integrins and growth factor receptors, GFR, with subsequent modification of GFR activity [2, 11]. It has been shown that the outcome of these interactions varies in different cell types, and depends on growth conditions. For example, in human epidermoid carcinoma HEp3 cells, α5 integrin binding to the epidermal growth factor receptor, EGFR, enhanced proliferation [10] while in Caco-2 and HT-29 colorectal carcinoma cells, α5/EGFR binding resulted in EGFR lysosomal degradation followed by proliferation arrest [6].

Alternatively, integrins may control cell fate via regulation of apoptosis, specifically anoikis, an anchorage-dependent apoptosis [12]. Intriguingly, the role of α5β1 integrin in regulation of anoikis appears to be controversial. In particular, up-regulation of α5β1 was essential for survival of MCF-10 breast carcinoma cells devoid of ECM substrate [13] whereas in human gastric carcinoma cells, hypoxia-inducible factor- mediated resistance to anoikis entirely depended on suppression of α5β1 integrin [8]. Therefore, studies addressing the role of individual integrins in different cell types are important for understanding the receptor-mediated regulation of mitogenic mechanisms in these cell types.

In the present study, we investigated the role of α5β1 integrin in proliferation of epidermoid carcinoma cells. We demonstrated that α5β1 regulates proliferation of these cells via twofold mechanism: by stimulating EGFR signaling cascade and by maintaining activated state of Akt kinase that is required for continuous suppression of apoptosis.

## RESULTS

### Down-regulation of α5β1 expression or inhibition of EGFR activity evokes similar effects on A431 cell proliferation but differ in regulation of cell survival

**Figure 1:**
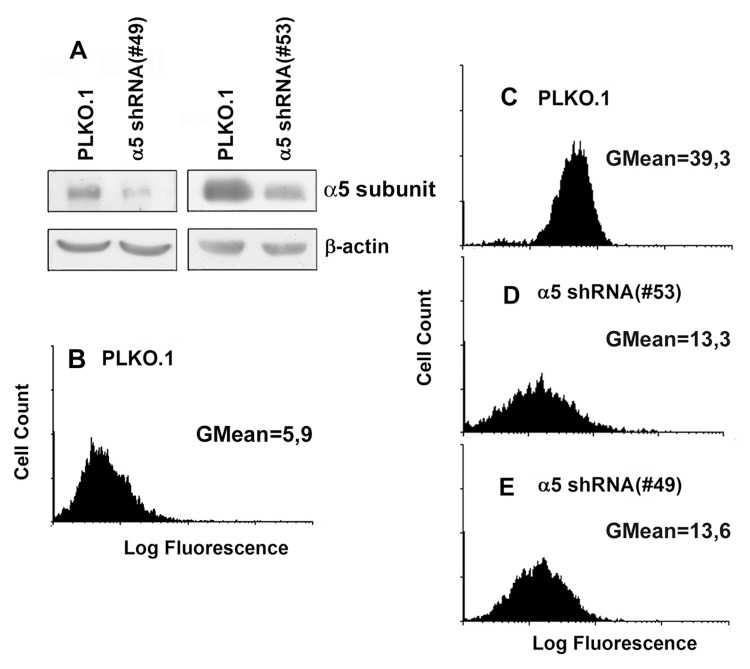
Efficient shRNA-mediated inhibition of a5 in A431 cells Cells were transduced with lentivirus, containing #49 or #53 PLKO.1 plasmid vectors with a5 shRNAs, or empty (control) vector as described in “Materials and methods”. (**A**) Western blot analysis of cell lysate proteins. The blots were probed with 1:1000 dilution of polyclonal α5 antibody and 1:500 dilution of β-actin mAb, stained using ECL system and scanned. (**B** and **C**) Flow cytometry of the cells tranduced with the empty (control) PLKO.1 vector and stained with FITC-labeled anti-mouse IgG (**B**) or with anti-α5β1 mAb and FITC-labeled anti-mouse IgG (**C**). (**D** and **E**) flow cytometry of the cells tranduced with the α5 shRNA PLKO.1 vectors #53 and #49, respectively, and stained as in **C**.

**Figure 2:**
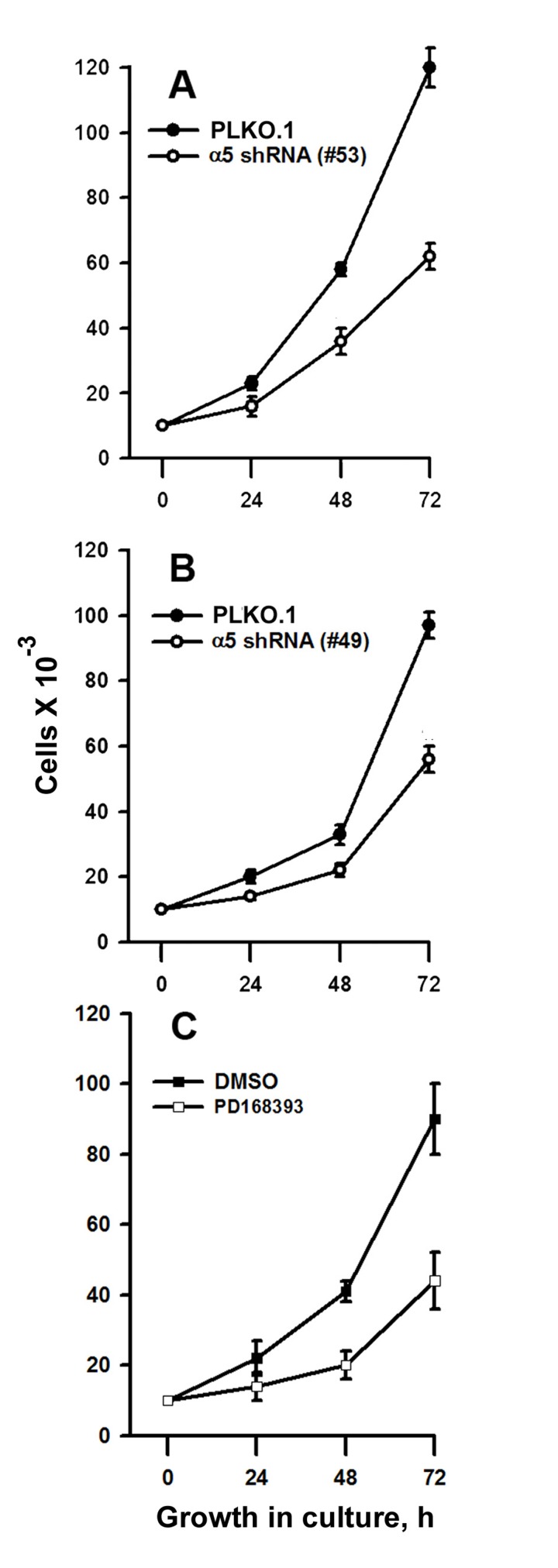
Integrin α5 knock-down or inhibition of EGFR suppress proliferation of A431 cells (**A** and **B**) Cells tranduced with control or α5 shRNA vectors were cultivated in serum-reduced medium for 24 h and seeded as described in “Materials and methods”. (**C**) Cells were cultivated in-serum reduced medium for 24 h, treated with 5 μM of the EGFR inhibitor, PD168393, at 37°C for 1 h, seeded in complete medium for 24-72 h and quantified as described in “Materials and method”. Control (untreated) samples contaned 0,5% DMSO. Shown are the mean ± SD of two independent experiments.

**Figure 3:**
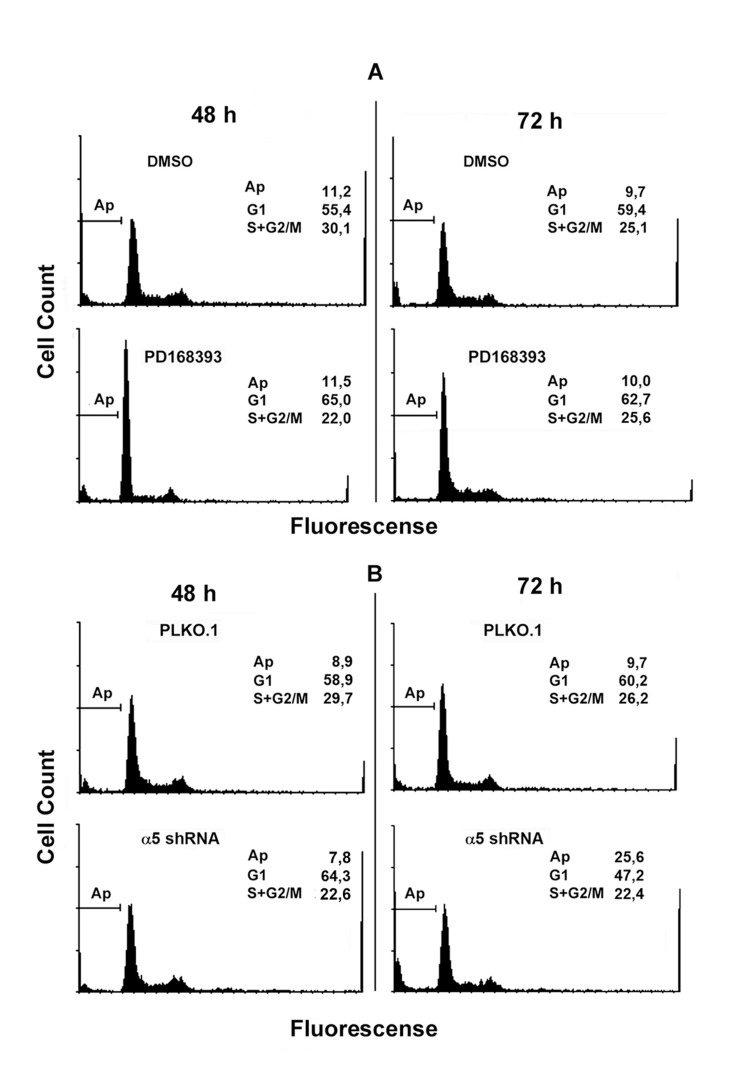
Integrin α5 knock-down and inhibition of EGFR exert different yet overlapping effects on cell cycle distribution of A431 cells Cells treated with PD168393 as detailed in Fig. 2 (**A**) or transduced with control or α5 shRNA-containing vectors (**B**) were seeded for 48-72 h, then fixed and prepared for flow cytometry as indicated in “Materials and methods”. FACS analysis was performed on a FACS-Calibur (Bekton Dickinson). Shown are typical experiments.

Synergistic effects on the mitotic activity of growth factor receptors, in particular EGFR, and integrins have been reported previously [2, 6, 11]. To elucidate the mechanisms of such synergy, we compared the effects on the proliferation of A431 cells of down-regulation of α5β1 and suppression of EGFR-mediated signaling. To this end, the kinase activity of EGFR was inhibited by commercially available inhibitor PD168393. The α5β1-mediated signaling was attenuated using siRNA technology. As shown in Figure 1, two different α5-specific shRNAs substantially decreased the amounts of α5 as was determined by immunoblotting or by detection of α5β1 expression on the cell surface using FACS-based technology. Down-regulation of α5β1 levels or inhibition of EGFR activity exerted similar negative effects on proliferation of A431 cell (Figure 2A-C). Statistically significant difference between the control and experimental groups was detected as soon as in 48 hrs. To further delineate inhibitory effects of a5β1 knockdown or EGFR inhibition, we performed analysis of the cell cycle progression using PI-based FACS. As shown in Figure 3, treatment with the EGFR inhibitor increased accumulation of cells in the G1-phase of the cell cycle and at the same time decreased their accumulation in the S- and G2/M-phases. Importantly, no cells with the sub-G1 amounts of DNA (a hallmark of the apoptotic DNA degradation) were detected in PD168393-treated cells. Thus, EGFR inhibition caused the G1 cell cycle arrest but no apoptosis. Interestingly, a5β1 knock-down resulted in the G1 cell cycle arrest 48 hours after infection with shRNAs and in substantial apoptosis starting 72 hours after infection that was evident by a significant increase of the sub-G1 population of cells. Therefore, inhibition of EGFR signaling led to the G1 proliferation arrest, whereas a5β1 knock-down resulted in the G1 cell cycle arrest and, at later time points, in apoptosis.

**Figure 4:**
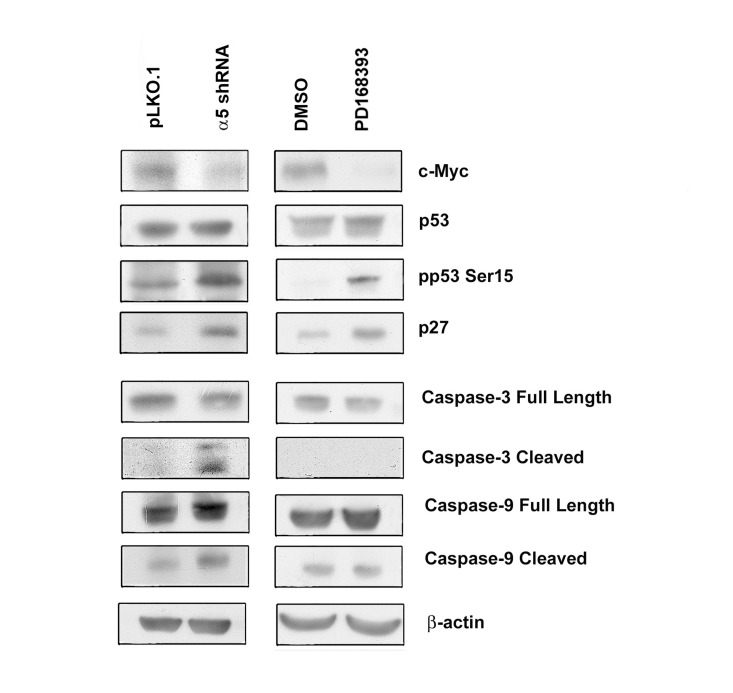
Integrin α5 knock-down and inhibition of EGFR differently affect expression of cell cycle and apoptosis regulators in A431 cells The cells were transduced with control or α5 shRNA-containing vectors or treated with PD168393 (as detailed in “Materials and methods” and Fig. 2) then western blot analysis and ECL-staining were performed. The blots were probed with 1:300 dulution of c-Myc antibodies and with 1:1000 dilution of other antibodies.

The cell cycle analysis data were confirmed by assessing expression of the proliferation and apoptosis markers in studied cell populations (Figure 4). Upon inhibition of EGFR or α5β1, expression levels of c-Myc decreased, whereas the active form of p53 and the cyclin dependent kinase inhibitor p27 increased. In contrast, inhibition of α5β1 but not EGFR resulted in activation of caspases 3 and 9 which correlated with the emergence of the sub-G1 peak in cells depleted of α5 integrin (Figure 3B).

### Signaling pathways that mediate effects caused by inhibition of α5β1 and EGFR

**Figure 5:**
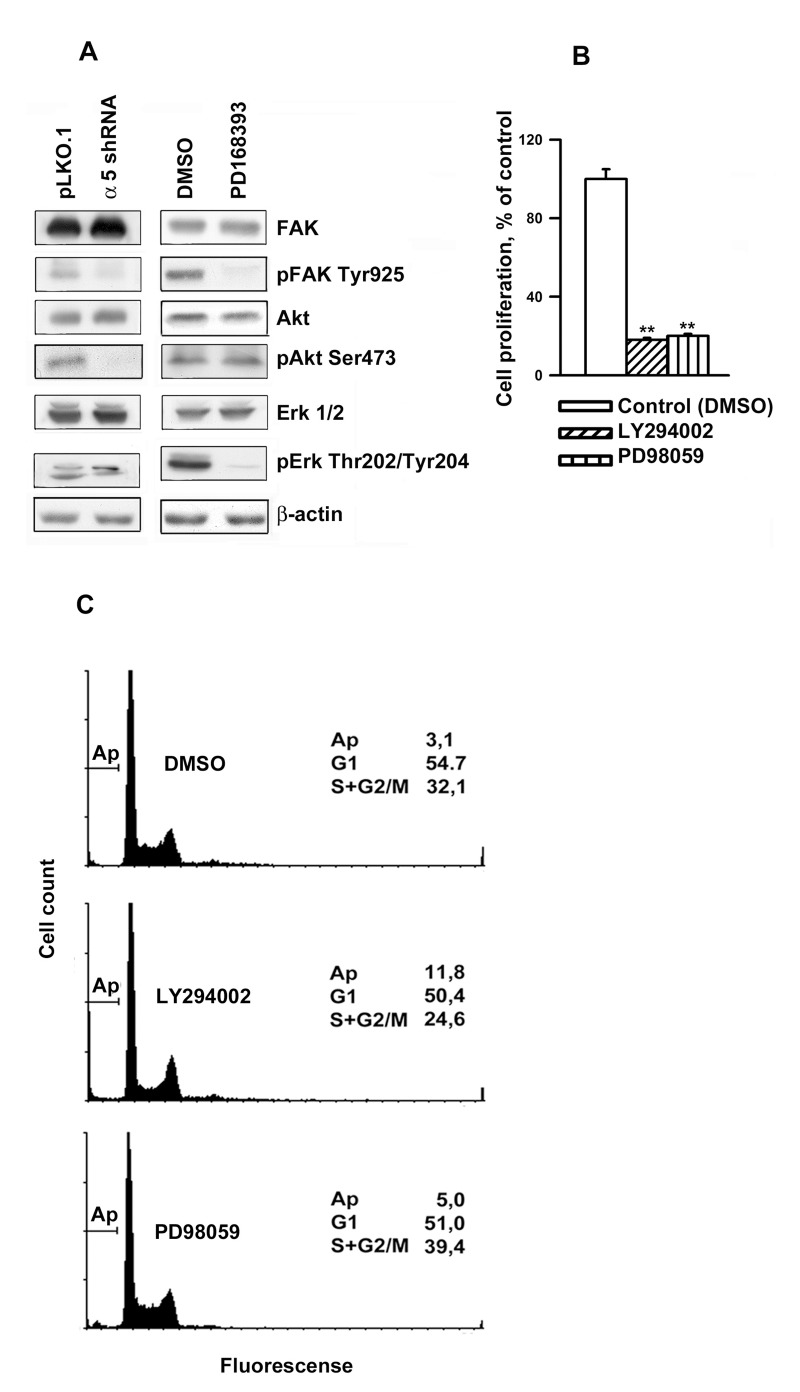
Integrin α5β1 and EGFR share common signal pathways in regulating A431 cell proliferation (**A**) Western-blot analysis of cell lysates proteins was perfomed as described in Fig. 4. The blots were probed with 1:1000 dilution of the listed antibodies. (**B**) Inhibition of Akt and Erk1/2 lead to a strongly reduced proliferation of A431 cells. Cells were incubated in serum-reduced medium, containing 25 μM of PI-3K inhibitor LY294002 (Sigma) or Erk inhibitor PD98059 (Calbiochem) or 0,5% DMSO, for 1 h and seeded in complete medium for 48 h whereupon the cells were harvested and quantified. (**C**) Inhibition of Akt (but not Erk1/2) induced significant apoptosis in A431 cells. Cells were treated as in indicated in (B), then fixed and prepared for flow cytometry as indicated in “Materials and methods”. FACS analysis was performed on a FACS-Calibur (Bekton Dickinson). Shown are typical experiments.

Next, we investigated possible common downstream signaling pathways for α5β1 and EGFR. We demonstrated that down-regulation of α5β1 or inhibition of EGFR activity substantially affected phosphorylation of focal adhesion kinase FAK at Tyr 925 (Figure 5A), that was reported previously to be required for FAK activity [14] . It has been shown that FAK co-localizes and physically associates with integrins at focal contacts and serves as an early sensor of integrin signaling [15]. The fact that inhibition of both α5β1 and EGFR led to the dramatic decrease of active FAK suggests that this enzyme is the primary common regulator for these signaling pathways.

Phosphatidylinositol-3-kinase (PI3K)/Akt and mitogen activated protein kinases (MAP), in particular, Erk, are known to transduce integrin signals [2]. Previously, we have demonstrated that α5β1 inhibition in the breast carcinoma cells caused a decrease in the activity of Akt and Erk2 [16]. We confirmed these effects in A431 cells (Fig. 5A), however, unlike a5β1 knock-down, attenuation of EGFR activity had no effect on the amount of active Akt, whereas the activity of Erk1 and Erk2decreased.To reveal whether this discrepancy accounts for the difference in cellular phenotypes induced by inhibition of α5β1 and EGFR, we tested the effects of Akt inhibitor LY294002 and MAP kinase inhibitor PD98059 on A431 cell proliferation. Figure 5B demonstrates that both treatments profoundly inhibited proliferation of these cells. These data are in line with highly reducedproliferation of various cancer cell lines resulted from the down-regulation of of PI3K/Akt signaling [1, 4]Importantly, unlike PD98059, prolonged treatment with LY294002 (up to 48 hours) induced significant apoptosis in A431 cells as was evident by FACS analysis (Figure 5C). Critical role of Akt in cell protection from apoptosis has been well documented [17, 18].

**Figure 6:**
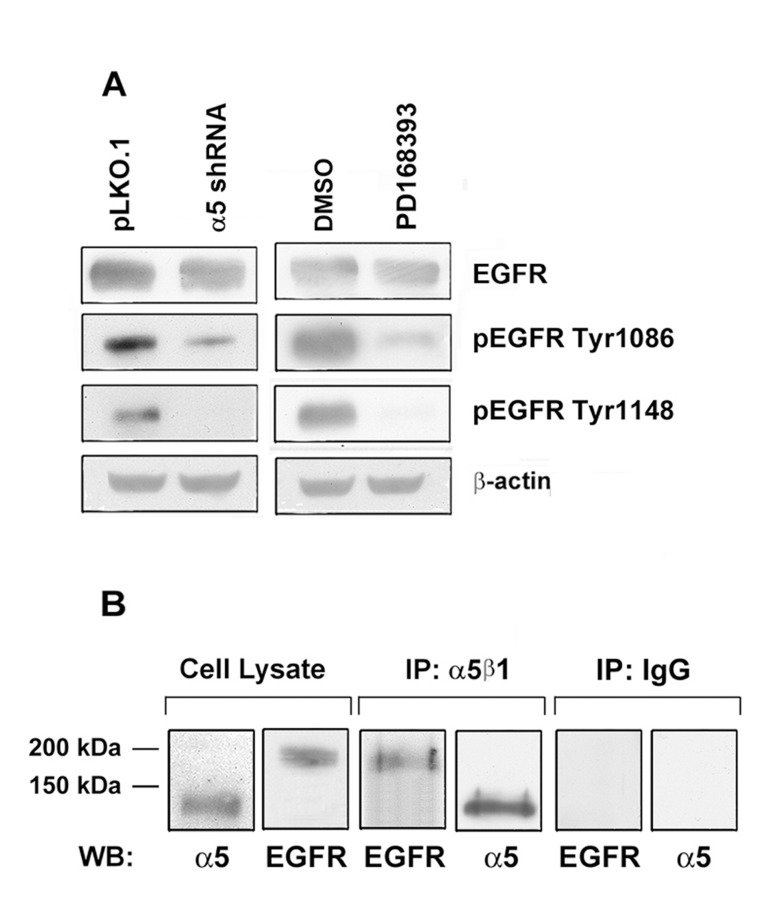
Integrin a5 knock-down strongly reduces the activity of EGFR in A431 cells (**A**) The cells were transduced with α5 shRNA or treated with PD168393 as described above followed by western-blot analysis. The blots were probed with 1:1000 dilution of EGFR and pEGFR antibodies and 1:500 dilution of anti-β-actin mAb. (**B**) Integrin α5 interacts with EGFR. Co-immunoprecipitation of EGFR with integrin α5. A 1 mg aliquat of cell extracts obtained from intact A431 cells was immunoprecipitated with 5 μl of the human α5β1 mAb (MAB 1999, Chemicon). The precipitates were run on SDS-PAGE and western blotted with the polyclonal antibodies to α5 subunit and EGFR. As a control, cell lysate proteins (30 μg) were resolved and western blotted with the α5 and EGFR antibodies. Control immunoprecipitation with an α5β1 isotype specific IgG is shown.

To verify whether decreased expression of a5β1partially or completely abolishes activation of EGFR, we studied phosphoryaltion status of EGFR at Tyr1086 or Tyr1148 in A431 cells depleted of α5. Phosphorylation at Tyr 1086 mediates matrix-activated α5β1 signaling whereas phosphorylation at Tyr 1148 occurs in response to epidermal growth factor [19, 20]. We demonstrated that indeed down-regulation of α5β1 caused significant decrease of both phosphorylated forms of EGFR (Figure 6A). Furthermore, we demonstrated that α5β1 and EGFR can be co-immunoprecipitated with anti-α5 antibodies (Figure 6B) thus suggesting physical interactions between these proteins in A431 cells.

## DISCUSSION

The role of α5β1 integrin in regulation of proliferation of normal and malignant cells is controversial. The expression of α5β1 was higher in proliferating retinal cells than in their quiescent counterparts [9]. Activation of α5β1 by its ligand fibronectin enhanced proliferation of cultured endothelium of lymphoid vessels [21]. Furthermore, growth rates of preadipocytes increased after the exogenous expression of α5β1 [22]. Likewise, the specific inhibitor of α5β1, JSM6427, caused retardation of proliferation of glioma [23] and endothelial [24] cells. On the other hand, inhibitory effects of α5β1 on cell proliferation have also been reported.Overexpression of α5β1 in Caco-2 and HT-29 colon cancer cells with low endogenous levels of this integrin abrogated their tumorigenicity [6]. Accordingly, it has been suggested that the inhibitory effects of tocopherol (vitamin E) on glioma cell proliferation were associated with higher expression of α5β1 [7]. It is conceivable that one of the reasons for these discrepancies is the cell type specific mechanisms of α5β1 signaling. Therefore, a detailed characterization of α5β1-mediated pathways in individual cell types emerges as an important question.

The results of our study agree with previous observations of growth stimulatory effect of this integrin [21-24]. Cooperative signaling between integrins and growth factor receptors has been demonstrated earlier [20, 25, 26]. Thus, interaction of α5β1 with growth factor receptors was shown to occur in the presence as well as in the absence of growth factors [19]. In Caco-2 intestinal epithelium cell line that lacks α5β1 the exogenous expression of this integrin induced cell proliferation, and this effect can be attenuated by the antibody that blocks EGFR activity [27]. These results are in line with our data that showed the decreased phosphorylation of FAK and Erk as a result of inhibition of α5β1 and EGFR signaling. These data are further corroborated by a dramatic decrease of two active phopshorylated forms of EGFR in cells with down-regulated α5β1 and by formation of complexes between α5β1 and EGFR (Figure 6).

Another mechanism of α5β1-mediated control of cell proliferation is associated with integrin-dependent maintenance of pro-survival signaling via continuous activation of Akt, a known suppressor of apoptosis. Data in Figures 3, 4 and 5 strongly suggest that α5β1 signaling is required for maintaining anti-apoptosis signaling in studied cells. In contrast, EGFR signaling in these cells is not involved in the control of cell survival.

In agreement with this notion is the fact that inhibition of Akt blocked α5β1-mediated pro-survival signaling in serum-deprived endothelial cells however, inhibition of MAPK had no effect on integrin-dependent anti-apoptosis [21].

In summary, our results suggest that α5β1 integrin regulates cell proliferation by at least two signaling mechanisms: maintaining pro-survival Akt-dependent signaling and activation of EGFR.

## MATERIALS AND METHODS

### Cell culture and chemicals

A431 human epidermoid carcinoma cell line was obtained from ATCC. Cells were cultured in DMEM supplemented with 10% fetal calf serum (HyClone, USA), 2 mM glutamine, 100 U/ml penicillin, and 100 μg/ml streptomycin, and incubated in a humidified incubator with 5% CO_2_ at 37°C. Erk and EGFR inhibitors, PD98059 and PD168393, resp, were purchased from Calbiochem (CA, USA), and other chemicals were obtained from Sigma (MO, USA).

### Lentiviral shRNA constructs

Bacterial glycerol stocks [clones NM_002205.1-2595s1c1 (#53) and NM_002205.1-1178s1c1 (#49) containing lentivirus plasmid vector pLKO.1-puro with shRNA specific for a5 integrin subunit was purchased from Sigma (MO, USA). The targeting sequences are: CGGCTCCTATATGTGACCAGAGTTCTCGAGAACTCTGGTCACATATAGGAGTTTTT (#53), CCGGCCATGATGAGTTTGGCCGATTCTCGAGAATCGGCCAAACTCATCATGGTTTTT (#49); pLKO.1-puro lentiviral vector without shRNA was used as a control. Lentiviruses were produced in HEK293T cells after co-transfection of lentivirus plasmid vector with shRNA or control vector with packaging plasmids using ExGen 500 Transfection Reagent (Fermentas). After 72 h medium containing lentivirus was collected and filtered through 0.45 uM filter. Filtered virus containing medium was used for cell infection. Cells were transduced with lentivirus in the presence of 8 mg/ml polybrene and selected with puromycin (1,0 mg/ml) for 4 days.

### Cell proliferation assay

Cells were cultivated for 24 h in serum-reduced medium and then seeded in DMEM supplemented with 10% serum at a density of 10^4^ cells/well into 48-well cell culture plates. After distinct time intervals, the cells were harvested and quantified using MTT-test.

### Cell cycle analysis

10^6^ cells were fixed with ice-cold 70% ethanol, resuspended in 1.0 ml solution of 40 μg/ml DNase-free RNase A in PBS and incubated for 30 min at room temperature. Propidium iodide was added to a fnal concentration of 20 μg/ml and cells were stored for 3 h on ice. Cell cycle analysis was performed by flow cytometry (FACS Calibur, BD) using WinMDI 2.8 software.

### Imunoblotting and immunoprecipitation

The following antibodies were used: anti-α5β1 integrin mAb and anti-α5 subunit polyclonal antibodies from Chemicon, anti-c-Myc mAb and anti-β-actin mAb from Santa Cruz Biotechnology; anti-p27 from BD; monoclonal antibodies to phospho-EGFR (Tyr1086/Tyr158), phospho-FAK (Tyr925), phospho-Akt (Ser473), phospho-Erk1/2 (Thr202/Thr204), c-Myc, caspase-3, caspase-9, phospho-p53 (Ser15) as well as polyclonal antibodies against EGFR, FAK, p53 were from Cell Signaling. Cells were extracted with RIPA buffer (1% Triton X-100 in 50 mM Tris-HCl pH 7.5, 150 mM NaCl, 0,5% DOX, 0.1% SDS), containing protease inhibitor cocktail (Sigma) and phosphatase inhibitor cocktail (Santa Cruz Biotech), each 1μl/10^6^ cells, and centrifuged at 13000 g for 10 min. 30 μg of cell lysate proteins were run on 7.5% SDS-PAGE under reducing conditions.

For immunoprecipitation experiments, 0.5 - 1 mg of proteins were immunoprecipitated with the appropriate antibody for 1 h at 4°C in the presence of 25 μl of protein A/G agarose (Amersham). Following SDS-PAGE, proteins were electroblotted onto a PVDF membrane. After reaction with specific primary antibodies the membrane was incubated with HRP-conjugated secondary antibodies, developed in ECL detection system (Amersham) and scanned.

### Statistical analysis

Statistical significance was determined with Student's test comparison between two groups of data sets.
